# Simultaneous Spectrophotometric Estimation of Haloperidol and Trihexyphenidyl in Tablets

**DOI:** 10.4103/0250-474X.65016

**Published:** 2010

**Authors:** S. P. Wate, A. A. Borkar

**Affiliations:** Sharad Pawar College of Pharmacy, Wanadongri, Hingna Road, Nagpur-441 110, India

**Keywords:** Haloperidol, trihexyphenidyl, simultaneous estimation, recovery study

## Abstract

The combination of haloperidol and trihexyphenidyl is a dosage form to be used as antidyskinetic agent. Literature revealed that there is no single method for the simultaneous estimation of these drugs in tablet dosage form, which prompted us to develop a simple, rapid, accurate, economical and sensitive spectrophotometric method. The simultaneous estimation method is based on the principle of additivity of absorbance, for the determination of haloperidol and trihexyphenidyl in tablet formulation. The absorption maxima of the drugs were found to be at 245.0 nm and 206.0 nm respectively for haloperidol and trihexyphenidyl in methanol and 0.1N HCl (90:10). The obeyance of Beer Lambert’s law was observed in the concentration range of 2.5-12.5 µg/ml for haloperidol and 1.0-5.0 µg/ml for trihexyphenidyl. The accuracy and reproducibility of the proposed method was statistically validated by recovery studies.

Haloperidol (HP) is an antidyskinetic and antipsychotic drug whose IUPAC name is 4-[4-(4-chlorophenyl)-4-hydroxy-1-piperidyl]-1-(4-fluorophenyl)-butan-1-one. Trihexyphenidyl (THP) is an antidyskinetic and antiparkinson drug whose IUPAC name is 1-cyclohexyl-1-phenyl-3-(1-piperidyl)-1-propanol. HP is official in BP [1] and THP in IP [2]. BP suggests a titrimetric assay method for HP, while IP suggest a titrimetric assay method for THP. Literature survey revealed that HPLC methods [3,4] have been reported for the estimation of HP and THP individually and with other drugs in pharmaceutical dosage forms. However, no method is reported for the simultaneous estimation of these drugs in combined dosage forms. This prompted us to develop simple, rapid, accurate, economical and sensitive spectrophotometric method.

Shimadzu 1700 UV/Vis spectrophotometer with matched cuvettes was used for the experimental work. The chemicals used were of analytical grade. Commercially available tablets of HP and THP in combination were procured from the local pharmacy. Standard HP and THP were received as gift samples from Stadmed Pvt. Ltd., Kolkata.

Standard stock solutions of HP and THP were prepared separately by dissolving 25 mg each of standard HP and THP in methanol and 0.1N HCl (90:10) and making up the volume to 50 ml with same solvent. Standard solutions (25 µg/ml) HP and THP were further prepared by taking 2.5 ml of stock solution of each drug in two 50 ml volumetric flasks separately and making up the volume to the mark with same solvent.

Overlain spectra of standard solutions of HP and THP were obtained and scanned between 200-300 nm (fig. 1). HP showed absorption maxima at 245.0 nm and THP showed at 206.0 nm. Calibration curve for each drug was prepared in the concentration range of 2.5-12.5 µg/ml for HP and 1.0-5.0 µg/ml for THP at corresponding wavelengths i.e. 245.0 nm and 206.0 nm. Amount of each drug was determined using simultaneous Eqn. as Cx= (A_2_ay_1_–A_1_ay_2_)/(ax_2_ay_1_–ax_1_ay_2_). Cy= (A_1_ax_2_–A_2_ax_1_)/(ay_1_ax_2_–ay_2_ax_1_), where, Cx= concentration of HP in g/100 ml, Cy= concentration of THP in g/100 ml, A_1_ = absorbance of laboratory mixture at 245.0 nm, A_2_ = absorbance of laboratory mixture at 206.0 nm, ax_1_ = absorptivity of HP at 245.0 nm, ax_2_ = absorptivity of HP at 206.0 nm, ay_1_ = absorptivity of THP at 245.0 nm and ay_2_ = absorptivity of THP at 206.0 nm. Percent estimation= (C×D)/W×100, where, C= Cx or Cy= concentration of HP or THP in g/100 ml, D= dilution factor and W= weight of drug (either HP or THP) in the laboratory mixture.

**Fig. 1:**
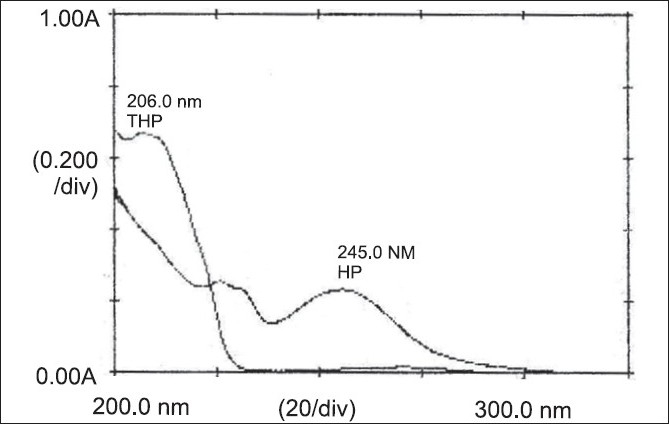
Overlain spectra of haloperidol and trihexyphenidyl Overlain spectra of haloperidol and trihexyphenidyl, solvent: methanol and 0.1 N HCL (90:10). λ_max_ of HP 245.0 nm, λ_max_ of THP 206.0 nm

Marketed tablets Halotex (Triton Health Care Pvt. Ltd., Chennai, India) were used for the simultaneous estimation of HP and THP. Twenty tablets were weighed and crushed to a fine powder. Powder equivalent to 50 mg of HP and 20 mg of THP (tablet contains 5 mg HP and 2 mg THP) was dissolved in the solvent and volume was made up to 50 ml. Insoluble excipients were separated by filtration. The filtrate was further diluted to get final concentration of both the drugs in the linearity range. Absorbance was noted at the selected wavelengths and percent label claim was determined by using the Eqn., Percent label claim= (Cx or Cy×D×W)/(Wm×L)×100 where, Cx or Cy= concentration of HP or THP in g/100 ml, W= average weight of tablet, Wm= weight of sample taken and L= label claim of sample taken.

Reproducibility, repeatability and accuracy of the proposed method were found to be satisfactory which is evident from the low values of standard deviation (SD), percent relative standard deviation (RSD) and standard error (SE) (Table 1). The accuracy and reproducibility of the proposed method was confirmed by recovery experiment, performed by adding known amount of the drugs to the preanalyzed formulations and reanalyzing the mixture by proposed method (Table 2). Percent recovery obtained indicates non-interference from the excipients used in the formulation. Thus, the method developed in the present investigation is found to be simple, sensitive, accurate and precise and can be successfully applied for the simultaneous estimation of haloperidol and trihexyphenidyl in tablets.

**TABLE 1:** RESULTS OF STATISTICAL DATA OF MARKETE FORMULATION

Tablet brand	Tablet component	Label claim (mg/tab)	SD	% RSD	SE
Halotex	Haloperidol	5	0.2484	0.2523	0.1110
	Trihexyphenidyl	2	0.2694	0.2662	0.1204

**TABLE 2:** RESULTS OF DRUG RECOVERY STUDY

Tablet brand	Amount of pure drug added (µg/ml)		% Recovery		SD	
	HP	THP	HP	THP	HP	THP
Halotex	2	2	100.28	98.76	0.0416	0.2538
	4	4	100.22	99.17		
	6	6	100.18	99.37		
